# PMADS: an integrated database of curated and proteomics-inferred associations between protein post-translational modifications and drug sensitivity

**DOI:** 10.1093/nar/gkaf1033

**Published:** 2025-10-16

**Authors:** Jie Zheng, Shuting Chen, Yinuo Zhang, Bingyue Zhang, Keying Qiao, Junbo Xu, Yuxiao Fan, Mengying Feng, Zhiyi Feng, Ziyan Wang, Haiyun Wang

**Affiliations:** Research Center for Translational Medicine, Shanghai East Hospital, School of Life Sciences and Technology, Tongji University, Shanghai 200092, China; Research Center for Translational Medicine, Shanghai East Hospital, School of Life Sciences and Technology, Tongji University, Shanghai 200092, China; Research Center for Translational Medicine, Shanghai East Hospital, School of Life Sciences and Technology, Tongji University, Shanghai 200092, China; Research Center for Translational Medicine, Shanghai East Hospital, School of Life Sciences and Technology, Tongji University, Shanghai 200092, China; Research Center for Translational Medicine, Shanghai East Hospital, School of Life Sciences and Technology, Tongji University, Shanghai 200092, China; Research Center for Translational Medicine, Shanghai East Hospital, School of Life Sciences and Technology, Tongji University, Shanghai 200092, China; Research Center for Translational Medicine, Shanghai East Hospital, School of Life Sciences and Technology, Tongji University, Shanghai 200092, China; Research Center for Translational Medicine, Shanghai East Hospital, School of Life Sciences and Technology, Tongji University, Shanghai 200092, China; Research Center for Translational Medicine, Shanghai East Hospital, School of Life Sciences and Technology, Tongji University, Shanghai 200092, China; Research Center for Translational Medicine, Shanghai East Hospital, School of Life Sciences and Technology, Tongji University, Shanghai 200092, China; Research Center for Translational Medicine, Shanghai East Hospital, School of Life Sciences and Technology, Tongji University, Shanghai 200092, China

## Abstract

Post-translational modifications (PTMs) are critical regulators of protein stability, localization, and function and have been increasingly implicated in shaping drug responses across diverse diseases. While numerous studies have reported PTM-related mechanisms of drug sensitivity or resistance, no resource has systematically catalogued these associations in disease-specific contexts. Here, we present protein modification and drug sensitivity (PMADS), an integrated database that systematically organizes PTM–drug–disease ternary associations, defined as PTM-centered drug responses in disease context. PMADS catalogues over 4700 curated associations manually extracted from biomedical literature and over 43 800 predicted associations derived from analysis of large-scale proteomics datasets. Together, PMADS covers >6000 proteins, 1000 drugs, 19 types of PTMs, and 300 disease classes. The database emphasizes both upstream regulatory mechanisms, such as drug-induced modulation of PTMs, and downstream effects, such as PTM-mediated changes in drug sensitivity. Each entry includes detailed annotations such as PTM site, effect description, ternary diagram, supporting evidence, confidence score, and cross-references to external databases, including PDB, PhosphoSitePlus, and DrugBank. PMADS features a user-friendly web interface with advanced search, interactive visualizations, and structured data export to support research in pharmacology, functional genomics, and precision medicine. PMADS is freely available at https://pmads-db.org.

## Graphical Abstract

**Graphical Abstract:**
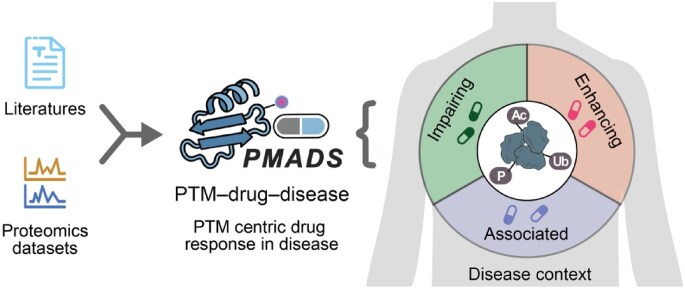

## Introduction

Post-translational modifications (PTMs) represent a fundamental layer of protein regulation, dynamically modulating protein stability, localization, and activity in response to cellular and environmental cues. Aberrant PTM patterns, such as dysregulated phosphorylation, acetylation, ubiquitination, or methylation, are increasingly recognized as hallmarks of disease progression, particularly in cancer, neurodegenerative disorders, and inflammatory conditions [1, 2]. PTMs not only contribute to the initiation and maintenance of pathological states but also critically influence therapeutic outcomes, including drug sensitivity, resistance, and toxicity [3–5]. For example, phosphorylation-dependent activation of signalling pathways confers resistance to targeted kinase inhibitors [6, 7], whereas histone acetylation status often predicts the response to epigenetic therapies such as histone deacetylase inhibitors [8]. These findings highlight the necessity of understanding PTM-mediated mechanisms in drug response to optimize treatment strategies and guide biomarker discovery.

Despite the accumulation of experimental evidence linking PTM dynamics to therapeutic outcomes, dedicated resources that systematically capture the ternary associations among PTMs, drugs, and disease contexts are lacking. Existing databases, such as PhosphoSitePlus [9], PTMD [10], dbPTM [11], PTMint [12], VPTMdb [13], UniProt [14] and Missense3D-PTMdb [15], provide crucial biological resources for researchers to understand PTM information and functions. These primarily focus on binary associations, emphasizing PTM–protein interactions or PTM–disease associations, thereby greatly aiding the elucidation of PTM biological functions (Supplementary Table S1). However, these resources do not explicitly integrate the ternary perspective necessary to elucidate how PTMs modulate drug efficacy or how drugs influence PTM states within disease-specific settings. This gap hinders efforts to comprehensively evaluate PTMs as actionable biomarkers or pharmacological targets in translational research.

To address this gap, we developed protein modification and drug sensitivity (PMADS), a freely accessible database that consolidates and catalogues both experimentally validated and computationally inferred PTM–drug–disease ternary associations. Here, we define a ternary association as a PTM-centered drug response in disease context, where PTM events may influence drug efficacy, drugs may alter PTM levels, or associations may be observed without a clear causal direction, all within a defined disease background. The curated dataset was generated by mining >2 million abstracts and full texts using a hybrid pipeline of natural language processing, keyword filtering, and expert validation, resulting in >4700 high-confidence associations. In parallel, the inferred dataset was derived from a systematic statistical analysis of large-scale proteomics data, providing complementary insights into potential associations. Together, PMADS encompasses 6420 proteins, 1037 drugs, 19 types of PTMs, and 300 disease classes. Each entry is annotated with the PTM type and site, drug and disease context, experimental system, source evidence, effect description, and confidence score, with cross-links to >20 external molecular and pharmacological databases. To ensure clarity and transparency, the web interface allows users to search and browse curated and inferred records independently. Through its structured design and user-friendly web interface, PMADS provides a valuable resource for exploring PTM-mediated mechanisms of drug action, facilitating hypothesis generation for biomarker discovery, target prioritization, and the advancement of precision medicine.

## Materials and methods

### Overview of data sources

The PMADS database systematically organizes PTM–drug–disease ternary associations, which we define as PTM-centered drug responses in disease context. This definition highlights that PTM events may modulate drug efficacy, drugs may alter PTM levels, or associations may be observed without a clear causal direction, all within the background of specific diseases.

**Figure 1.**
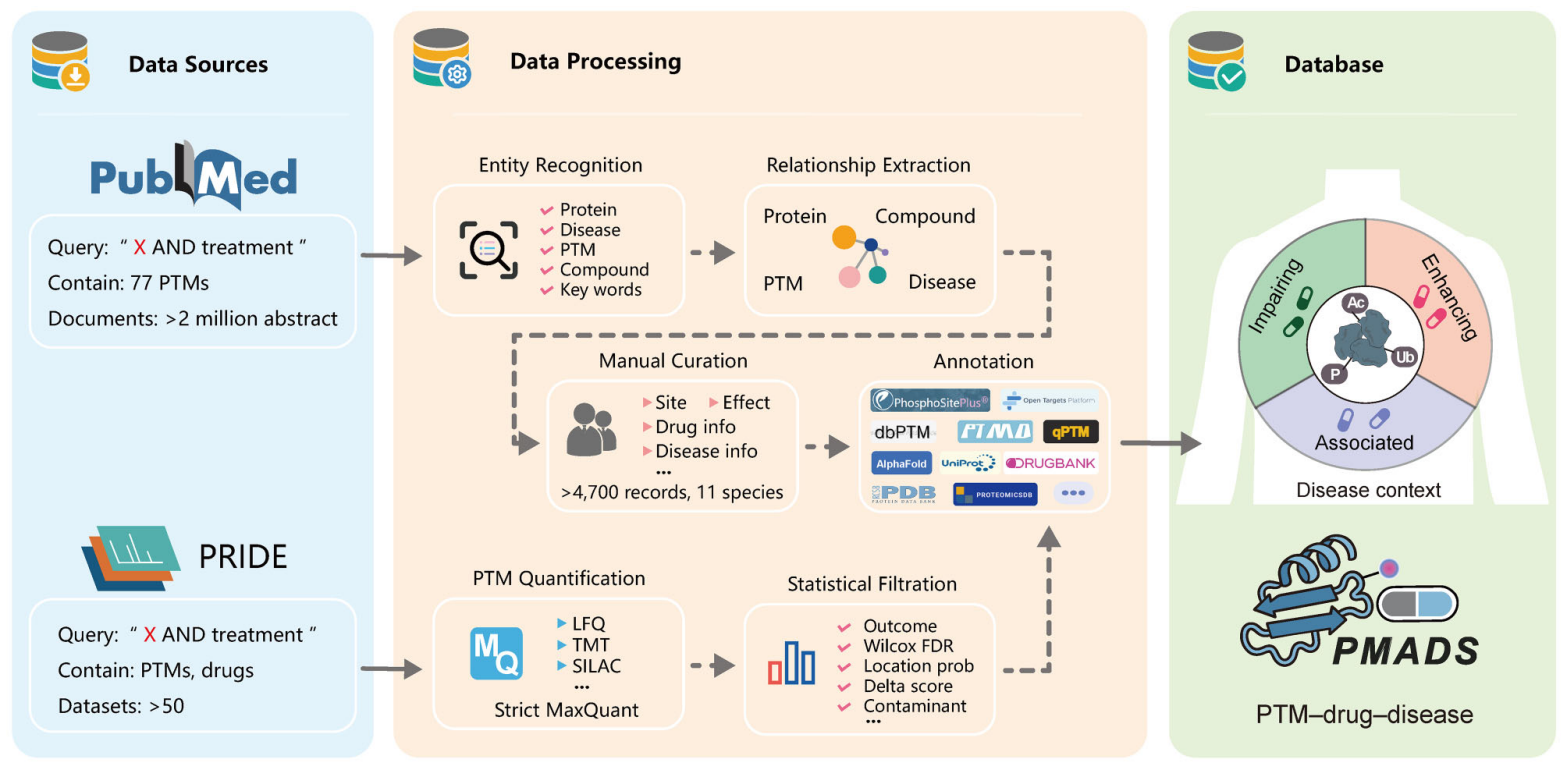
PMADS workflow. PMADS integrates PTM–drug–disease associations from curated literature and inferred proteomics datasets. Curated literature pipeline: PubMed queries (‘X AND treatment’, 77 PTMs) yielded >2M records. Named entity recognition extracted proteins, diseases, PTMs, compounds, and keywords, followed by relationship extraction to identify protein–compound–PTM–disease links. Manual curation captured site, effect, drug, and disease metadata (>4700 entries, 11 species), which were enriched using PhosphoSitePlus, dbPTM, qPTM, AlphaFold Protein Structure Database, UniProt, PDB, DrugBank, Open Targets, ProteomicsDB, and others. Inferred omics pipeline: PRIDE datasets were processed via strict MaxQuant quantification (LFQ, TMT, SILAC). Multi-criteria filtering included Wilcoxon test with false discovery rate (FDR) correction, site localization probability, delta score, and contaminant removal. Filtered associations were annotated with the same resources as the curated pipeline to yield a unified, high-confidence PMADS dataset. Each entry is classified into one of three regulatory classes (Enhancing, Impairing, Associated).

PMADS integrates two complementary types of PTM–drug–disease associations: (i) curated associations, manually extracted from >2 million biomedical abstracts via a structured text-mining and rule-based curation pipeline and (ii) inferred associations, computationally derived from large-scale public proteomics datasets through statistical analysis of drug-induced PTM alterations (Fig. 1). These two datasets are processed through distinct pipelines and maintained separately in the database, with a *Status* field indicating the source (curated or inferred) for each record.

### Document acquisition and entity recognition

To systematically capture the literature describing how PTMs fluence therapeutic responses, we first compiled a comprehensive lexicon of 77 PTM types from dbPTM [11], covering both common (e.g. phosphorylation, acetylation, ubiquitination, methylation) and rare (e.g. sulfation, nitration, glycation, geranylgeranylation) PTMs. This PTM-specific lexicon was used to construct search queries for large-scale text mining, resulting in the retrieval of >2 million biomedical abstracts (Fig. 1).

Next, entity extraction was performed using PubTator, a widely used biomedical text-mining tool employing AI models for semantic and relational tagging of proteins, chemicals, diseases, and genetic variants [16]. Proteins, diseases, and drugs terms were identified using PubTator annotations. PTM entities were extracted using a custom string-matching approach supplemented by the NCBI TranslationSet, which generates inflectional and derivational variants (e.g. ‘phosphorylation’ expanded to ‘phosphorylations’, ‘phosphorylates’, and ‘phosphorylating’).

To identify regulatory associations and mechanistic descriptions, we built a curated keyword corpus inspired by the HIT database [17] and augmented it with manually extracted domain-specific terms. This initial set of 28-base keywords (e.g. ‘increase’, ‘decrease’, and ‘promote’) was expanded via the TranslationSet, resulting in >210 context-aware keyword variants for downstream pattern matching. To ensure consistency, all matched keywords were normalized and mapped back to the original 28-base keywords.

### PTM–drug–disease ternary relation extraction

While keyword identification provided an initial filter for capturing regulatory contexts, sentences frequently contained multiple regulatory terms, and simple lexical co-occurrence was insufficient to determine the precise directionality or nature of PTM–drug–disease relationships. To refine this process, we first performed dependency parsing using Stanza [18] to capture the syntactic relationships among entities. On the basis of these parse trees, we applied a manually curated rule set to extract and validate the PTM–drug–disease relationships, ensuring accurate linkage. For example, this approach allows the system to distinguish between general descriptions (e.g. ‘phosphorylation is a modification of a protein’) and mechanistic interactions (e.g. ‘phosphorylation of EGFR confers resistance to gefitinib’).

The extracted relationships were then manually reviewed and mapped to one of the three regulatory classes (stored in the *Regulatory Class* field of the database) on the basis of the contextual meaning of the effect terms and evidence sentences:

- Enhancing: PTM events that support or improve drug efficacy. This includes cases where the presence or upregulation of a PTM enhances the therapeutic effect of a drug or, conversely, where PTM loss or downregulation reduces drug efficacy.
- Impairing: PTM events that reduce or antagonize drug efficacy. This includes cases where the presence or upregulation of a PTM impairs the drug’s therapeutic effect or, conversely, where PTM loss or downregulation restores or improves drug efficacy.
- Associated: Indicates that a PTM–drug link is present, but the mechanistic directionality is unclear or cannot be inferred with confidence. This includes instances where PTMs are altered in response to drug treatment or when textual evidence suggests an association without specifying whether the effect is enhancing or impairing.

This classification reflects both the causal direction (PTMs acting on the drug response versus the drug altering PTMs) and the clarity of mechanistic interpretation, enabling users to distinguish interpretable regulatory effects from ambiguous associations.

To help users intuitively interpret ternary associations, we provide a cartoon visualization named the *Ternary Diagram* field for each curated record. In these diagrams, PTM, drug, and disease are colour-coded (pink, grey, and blue, respectively), and the regulatory flow is indicated with directional arrows. Although we do not explicitly categorize ternary associations into distinct mechanistic paths, the visual design loosely reflects one of the three common patterns: (i) PTMs modulate drug efficacy in disease, (ii) drugs perturb PTM levels that affect disease outcomes, or (iii) drugs influence both PTM alterations and disease progression (Supplementary Fig. S1). This structured representation enables users to identify both simple and multilayered regulatory mechanisms underlying PTM-mediated drug sensitivity.

Each curated record was assigned a confidence score ranging from 2 to 5, quantifying the clarity and strength of the association. Scores of 0 were used internally to denote cases with no clear association and were therefore not included in the database. For user convenience, scores are further mapped to categorical confidence levels (High, Moderate, Low). The detailed scoring rules are provided in Supplementary Table S2.

### Inferred associations from public proteomics datasets

To complement manually curated literature-based associations, we integrated inferred PTM–drug–disease associations derived from a systematic analysis of large-scale public proteomics datasets. Specifically, we processed 50 proteomic datasets from the PRIDE Archive [19], a public repository of mass-spectrometry-based experiments maintained by the European Bioinformatics Institute. Peptide and PTM quantification were performed using MaxQuant. To ensure high data quality, we retained only PTM sites with a MaxQuant score >40, localization probability >0.8, and delta score >40, while excluding potential contaminants and reverse sequences. Differential PTM abundance upon drug treatment was assessed using non-parametric Wilcoxon tests. PTM changes with an adjusted *P* < 0.05 were considered statistically significant (confidence score = 5). In addition, PTM sites with *P* < 0.05 and an absolute log_2_ fold change >10, but derived from datasets with limited sample sizes, were retained as low-confidence entries (confidence score < 5). Entries retained in the database have scores ranging from 3 to 5, corresponding to differential, relatively significant, or significant associations based on *P*-value and fold-change thresholds. As with curated data, scores are also mapped to categorical confidence levels (High, Moderate, Low), summarized in Supplementary Table S2. Inferred PTM–drug–disease associations were identified from experimental systems where drug exposure induced significant alterations in PTM levels in a disease-relevant context. Each inferred association is annotated with the same annotations of the reviewed association and is labelled ‘inferred’ in the *Status* field of the database for transparency. These associations offer hypothesis-generating value and complement experimentally validated literature findings.

### System architecture and web implementation

The PMADS database was implemented using Django 5 as the primary web framework and is hosted on an Apache2 server. Data are stored and managed in a MySQL relational database, ensuring scalability and efficient querying. The front end integrates multiple interactive and visualization frameworks, including ECharts for dynamic charting, jQuery for interactive components, and Mol* (Molstar) for molecular structure visualization.

To improve its content and contextual scope, PMADS integrates curated data and annotations from several high-quality resources, including OpenTargets [20], CPTAC [21], PTMD [10], DecryptM [22], and FunScoR [23]. These datasets collectively provide complementary evidence for the relevance of PTMs in disease, molecular pathways, and therapeutic contexts, enhancing the use of PMADS as a comprehensive research platform.

PMADS offers flexible and multilayered access to its curated content. A quick aggregate search enables rapid retrieval of records by protein, gene, PTM site, or drug. An advanced search interface allows users to construct customized queries with multiple parameters for precise filtering of PTM–drug–disease associations. A browse module supports the systematic exploration of all entries in the database, while comprehensive help documentation facilitates user onboarding. To support computational workflows, PMADS also offers an application programming interface (API) end-point for programmatic access to curated data.

In addition, PMADS provides extensive cross-references to a wide spectrum of external molecular, structural, and pathway resources, enabling users to contextualize each PTM–drug–disease relationship. These include protein-centric databases such as PDB [24], AlphaFold Protein Structure Database [25], PDBbind [26], UniProt [14], SCOP [27], CATH [28], PANTHER [29], and the Human Protein Atlas [30]; PTM-focused repositories such as PhosphoSitePlus [9], dbPTM [11], qPTM [31], PTMcode2 [32], CPLM [33], and PTMD [10]; drug-related datasets such as TTD [34], DrugBank [35], ChEMBL [36], BindingDB [37], PharmExCloud (https://data.pharnexcloud.com/), GDSC [38], and DecryptM/E [22]; and disease- and network-oriented resources such as CTD [39], DMRdb [40], STRING [41], BioGRID [42], Gene Ontology (GO) [43], and KEGG [44]. Through this integration (Supplementary Table S3), PMADS allows users to seamlessly access molecular details, structural insights, and functional annotations relevant to each curated association, supporting downstream analyses in drug discovery and disease research.

## Results

### Database content overview

**Figure 2.**
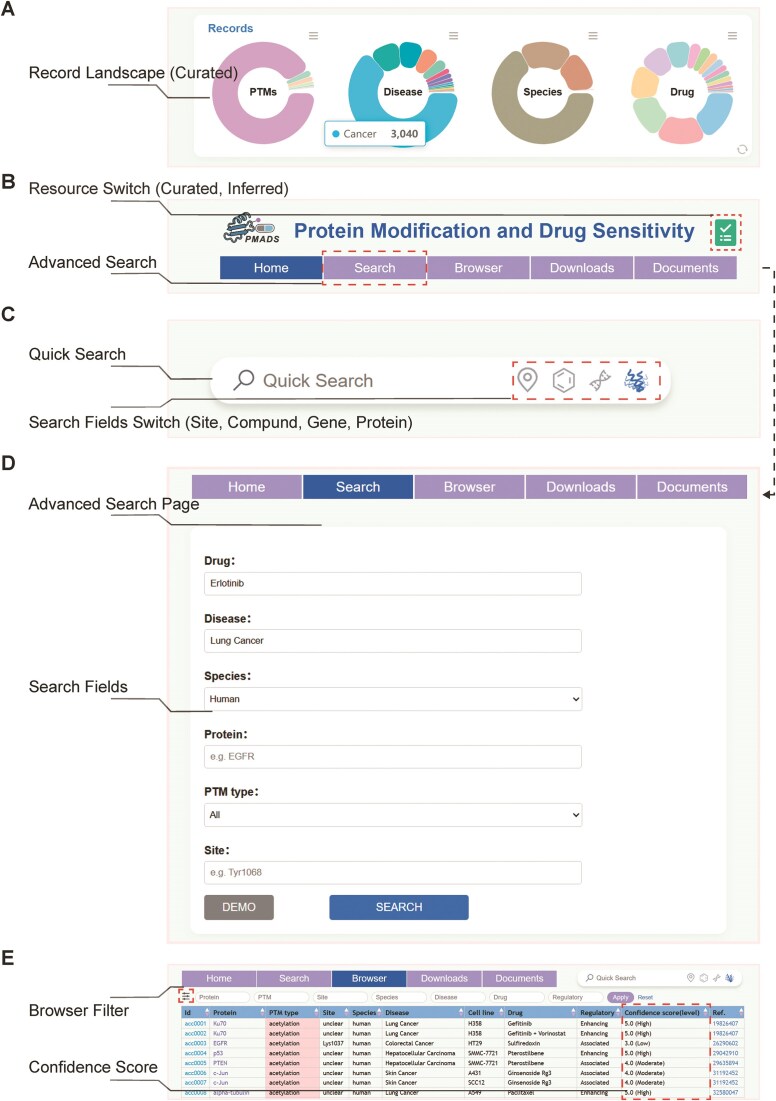
PMADS usage. (**A**) PMADS-curated record landscape. The database home page displays the distribution of PTMs, diseases, drugs, and species via pie charts. These charts are interconnected; clicking any sector of any pie chart reveals associations across other categories—e.g. viewing PTM classifications within cancer. (**B**) Navigation bar and resource switching. The database provides fundamental navigation functions including search, browse, download, and help documentation. Users can click the icon in the upper-right corner of the navigation bar to switch between curated and inferred records at any time, enabling corresponding data retrieval and browsing. (**C**) Quick search entry. A composite quick search entry is provided on the right side of the database page’s navigation bar. Users can perform rapid database searches by selecting PTM sites, compounds, genes, and proteins. The search bar offers auto-complete functionality for user convenience. (**D**) Advanced search page. The search page enables multi-field combination searches across drug, disease, species, protein, PTM type, and site. For example, search for all records of Erlotinib in human lung cancer. (**E**) Browse page. This page allows quick viewing of summary information for all database records, including record ID, PTM type, drug, disease, confidence score, reference source, and more. Users can further filter records of interest by clicking the filter button below the navigation bar.

PMADS hosts a comprehensive collection of curated PTM–drug–disease associations derived from biomedical literature mining. As of July 2025, the current release of PMADS comprises 4775 manually curated PTM–drug–disease associations, spanning >500 proteins, 1022 drugs, ∼1000 modification sites, and ∼300 disease classes across 11 species (Fig. 2A and Supplementary Table S4). These records include 19 PTM types, with phosphorylation being the most frequently observed, followed by acetylation, ubiquitination, and methylation.

The ternary diagrams accompanying each record reflect three common regulatory patterns: PTM changes influencing drug efficacy in a disease context (20.80%), drugs modulating PTMs that subsequently affect disease outcomes (76.31%), and drugs simultaneously regulating PTM states and disease progression (2.89%). Among these records, 63.66% are related to cancer, reflecting the dominant focus of PTM research being on oncology, while the remainder cover inflammatory, metabolic, and infectious diseases.

Across the curated dataset, PMADS integrates diverse species sources (human, mouse, rat, etc.) and experimental systems (cell lines, tissues). Supplementary Table S4 summarizes the distribution of associations by disease class, highlighting prevalent representations of cancer, metabolic disorders, and cardiovascular diseases. Certain kinases (e.g. AKT1 and ERK1) are disproportionately represented, which is consistent with their therapeutic relevance.

In addition, PMADS includes a separate module of predicted associations derived from large-scale public proteomics datasets, which are annotated as ‘inferred’ and described in a dedicated section below.

### Database utility

#### Overview of access and functionality

PMADS offers a robust yet user-friendly interface to facilitate exploration of PTM–drug–disease associations. On the database home page, a series of pie charts provides an overview of the database’s coverage across PTM, disease, species, and drug categories (Fig. 2A). Quick switching between data sources (curated and inferred) can be done by clicking the icon in the upper-right corner of any page (Fig. 2B). A quick search bar positioned at the top right of the home page enables instant retrieval of records by protein name, gene symbol, PTM site, or drug name (Fig. 2C). For more complex queries, the standard search allows filtering by multiple parameters, such as drug name, disease class, species, protein name, PTM type, and PTM site (Fig.2B and D), supporting combinatorial searches (e.g. ‘PTMs of Erlotinib + Human + Lung cancer’).

A browse module is used to systematically explore database entries grouped by protein name, PTM type, PTM site, disease class, drug name, etc. Users can view and filter sortable tables by choosing specific fields (Fig. 2E). Each record page presents regulatory class, ternary diagram, evidence sentences, confidence score, and links to external databases (e.g. PDB and DrugBank).

Data export is supported via downloadable ZIP formats. In addition, interactive visualization is integrated throughout: ECharts is used to display summary charts by PTM type, disease class, and species type and Mol* enables users to view structural context when PTM sites correspond to resolved protein structures. The *Ternary Diagram* for each record visually illustrates the PTM–drug–disease regulatory flow using colour-coded nodes and directional arrows, enhancing intuitive interpretation. A dedicated document section offers an overview of the database, a detailed user guide, and frequently asked questions to help users understand the platform’s structure and functionality.

#### Case example: ERK1 pThr202 with CUDC-907 in pancreatic cancer

**Figure 3.**
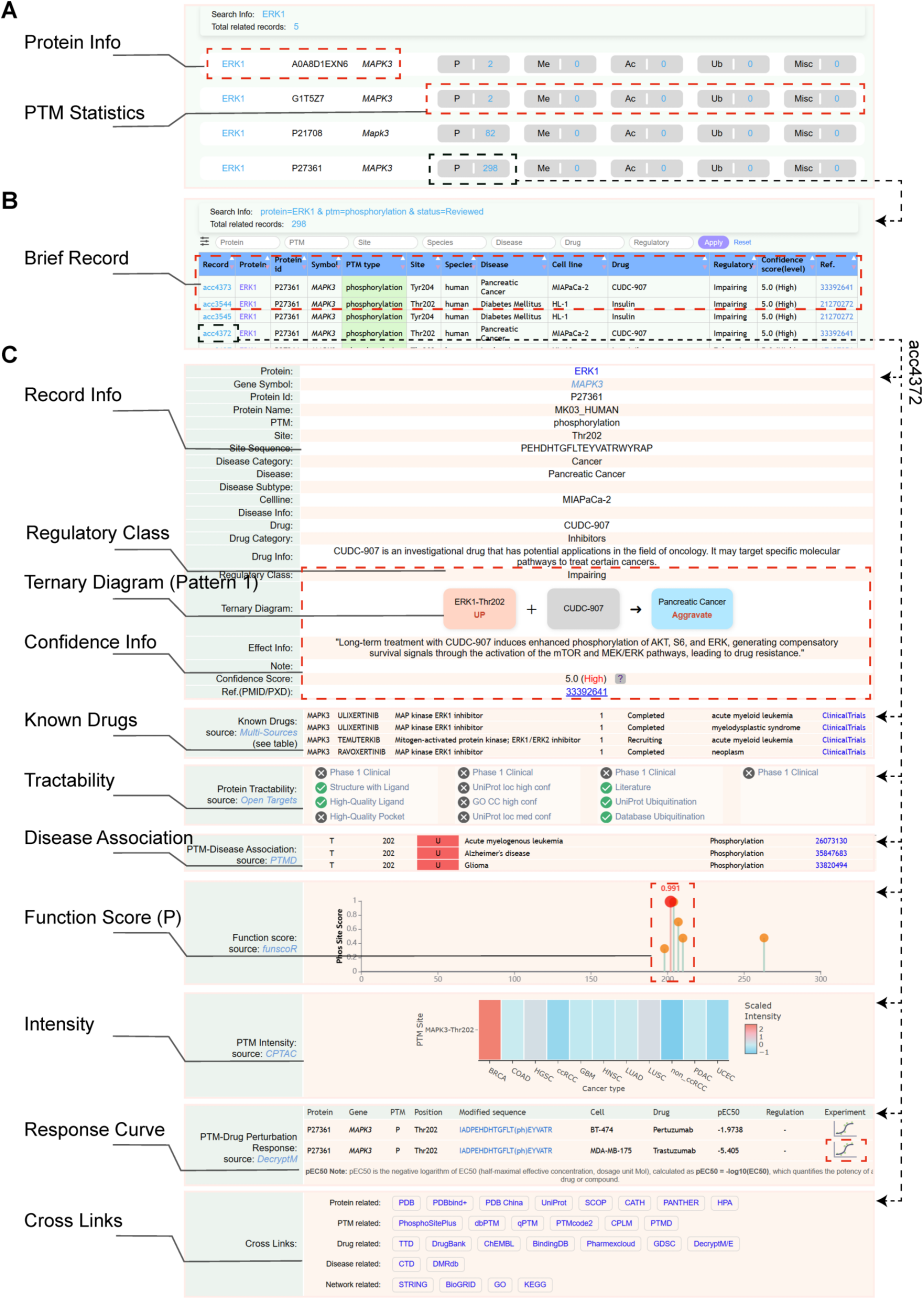
PMADS web interface. (**A**) Quick search results by protein (ERK): summary of proteins and corresponding PTM counts identified for each protein. (**B**) Detailed search results for ERK phosphorylation: breakdown by PTM information, drug, disease class, and effect description for phosphorylation entries. (**C**) Record viewer: curated details for a selected PTM–drug–disease association, including known drugs, disease association, PTM intensity visualization, function score, response curve, and cross‐links to external databases.

To illustrate the utility of PMADS, we highlight an example involving ERK1 phosphorylation at Thr202 and its impact on CUDC-907 sensitivity in pancreatic cancer. Using a quick protein search for ERK1 (UniProt ID: P27361), users can access a summary of all curated PTMs for this protein, including phosphorylation (298 entries), methylation (0), acetylation (0), ubiquitination (0), and other modifications (0) (Fig. 3A).

Upon selecting the phosphorylation category, users can further examine site–specific associations (e.g. Thr202 and Tyr204) with various drugs, such as CUDC–907 and imatinib, across multiple disease contexts, including pancreatic cancer and leukaemia (Fig. 3B). Each record summarizes the PTM–drug–disease relationship, including effect direction and an associated confidence score. For instance, entry acc4372 describes a PTM–drug–disease relationship involving ERK1 pThr202 and Thr202. It is annotated as the *Regulatory Class* ‘Impairing’, indicating that upregulation of ERK1 phosphorylation at Thr202 reduces the therapeutic efficacy of CUDC–907 in pancreatic cancer.

This entry includes experimental evidence showing that inhibition of ERK1 phosphorylation markedly reduces the anti-proliferative effect of CUDC–907 in MIAPaCa–2 cells (Fig. 3C) [45]. The record provides detailed annotations, including protein information, PTM site and sequence, drug and disease context, regulatory class, ternary diagram, effect information, confidence score (5.0), source sentence, species, and the corresponding ternary diagram.

#### Functional exploration and cross-reference capabilities

In addition to individual records, PMADS integrates multidimensional data to contextualize PTMs within broader therapeutic landscapes. Specifically, users can

- visualize PTM sites in 3D protein structures and examine the spatial location on solvent-exposed surfaces, binding pockets, or buried regions [25];
- assess target tractability by referencing associated drugs and known targetability metrics [20];
- survey PTM regulation across disease contexts, distinguishing upregulation versus demodification patterns [10];
- review functional importance scores for phosphorylation sites, such as ERK1 Thr202, which have high scores, suggesting critical biological roles [23];
- explore the abundance profiles of PTMs across tumour types, providing insights into potential PTM-based predictive biomarkers of drug sensitivity [46];
- investigate dynamic PTM responses under drug perturbations, such as upregulation of ERK1 Thr202 phosphorylation upon treatment with pertuzumab or trastuzumab [22];
- leverage extensive cross-references to external resources, including UniProt, PhosphoSitePlus, DrugBank, and GO, for enriched functional, structural, and pharmacological annotations.

Together, these features facilitate hypothesis generation for combination therapies and predictive biomarker discovery. For instance, PMADS entries such as acc0137 and acc0397 highlight how PTM modulation enhances the efficacy of imatinib and cisplatin, respectively [47, 48].

### Inferred association highlights

In addition to manually curated literature-based associations, PMADS incorporates a complementary set of predicted PTM–drug–disease associations inferred from systematic analyses of large-scale public proteomics datasets. Specifically, we processed 50 proteomics datasets from the PRIDE Archive and applied MaxQuant for peptide identification and PTM site quantification. PTM-level differential analysis was then conducted using non-parametric Wilcoxon tests to identify statistically significant changes upon drug treatment in disease-relevant samples.

To ensure transparency and avoid conflation with curated records, these predicted associations are labelled ‘inferred’ in the *Status* field and stored in a separate module. The current release includes >43 800 inferred associations spanning 6080 proteins, 22 362 PTM types, 39 drugs, and 18 disease types, with a primary focus on cancer. Notably, phosphorylation accounts for the majority of inferred modifications, reflecting its prevalence and detectability in large-scale proteomics. These results substantially expand the coverage of PMADS beyond literature-derived entries and offer new mechanistic hypotheses for drug response modulation.

For instance, in tamoxifen-resistant MCF7 cells (dataset PXD001812), Tyr15 phosphorylation of Cyclin-dependent kinase 1 (CDK1) is elevated relative to drug-sensitive controls (log_2_ fold change >20, acc06739). Tyr15 is an inhibitory site, phosphorylated mainly by WEE1 and PKMYT1, that delays CDK1 activation and mitotic entry. Increased pTyr15 has been linked to altered cell-cycle control and endocrine therapy resistance [49]. Similar patterns occur in gefitinib-resistant lung cancer models, where WEE1 upregulation increases Tyr15 phosphorylation and promotes resistance [50]. These findings support the biological plausibility of the inferred records in PMADS.

## Discussion

PTMs have emerged as critical regulators of drug response across diverse diseases, particularly in oncology and immune disorders. Despite the increasing availability of experimental evidence, no existing resource has systematically catalogued ternary PTM–drug–disease associations. PMADS addresses this gap by integrating >4700 experimentally supported associations identified through large-scale literature mining, entity recognition, and rigorous manual curation. In addition, PMADS incorporates an independent module of 43 888 inferred associations derived from large-scale public proteomics datasets, expanding coverage beyond the published literature. This hybrid approach balances coverage and reliability by combining curated and inferred data, offering a comprehensive foundation for exploring PTM-mediated mechanisms of drug action and resistance.

An additional challenge lies in the fact that many inferred associations are correlative rather than strictly causal, which may complicate user interpretation and prioritization. To mitigate this, PMADS provides a multilayered annotation framework. Each entry is labelled by evidence type (curated or inferred), assigned both a numerical confidence score and a categorical confidence level (High, Moderate, Low), and classified into one of three regulatory classes (Enhancing, Impairing, Associated). This design ensures that curated, high-confidence Enhancing or Impairing entries can be prioritized as the most reliable biological evidence, whereas Associated or low-confidence inferred entries are explicitly presented as hypothesis-generating leads. Nevertheless, several limitations remain. Firstly, the current pipeline primarily extracts candidate associations from abstracts using conventional entity recognition. While abstracts often highlight core findings, they may omit mechanistic nuances or contextual evidence present only in full texts. To mitigate this, all candidate records underwent full-text review by two independent curators. Future versions of PMADS will incorporate large language models (LLMs) for full-text analysis and semi-automated validation, which is expected to increase recall without compromising precision.

Secondly, establishing reliable ternary PTM–drug–disease relationships remains inherently challenging. While existing databases such as PTMD or PhosphoSitePlus offer curated binary interactions (e.g. PTM–disease), PMADS extends beyond ternary associations with three common regulatory patterns: (i) PTMs modulating drug efficacy, (ii) drugs modulating PTMs that subsequently affect disease outcomes, and (iii) complex bidirectional or feedback interactions. These regulatory patterns improve interpretability but cannot yet fully capture dynamic or time-dependent mechanisms, such as the emergence of PTM-driven drug resistance during chronic therapy.

Thirdly, PMADS currently employs a record-centric architecture, with each entry capturing a unique PTM–drug–disease instance. While this preserves the biological context, it may hinder higher-level data synthesis. To address this, the database supports protein-, site-, and PTM-level searches and continues to refine its interface to improve navigation and data synthesis.

Looking ahead, PMADS is positioned to evolve into a more predictive and integrative platform through the incorporation of full-text mining, LLM-assisted annotation, and protein-site-level drug sensitivity prediction using multi-omics data. In parallel, we will substantially expand the inferred data by incorporating analyses from a larger number of PRIDE Archive, thereby enhancing the coverage of PTM–drug–disease association discovery. Notably, PTM-Mamba, a novel protein language model that uniquely encodes both wild–type and post-translationally modified sequences, offers significant potential for PTM-specific functionality modelling and druggability inference [51]. By integrating PTM-Mamba into future PMADS workflows, we aim to enable quantitative evaluation and prediction of PTM–drug–disease associations—such as by estimating the impact of a modification on drug efficacy or inferring potential PTMs that affect therapeutic response. Ultimately, these enhancements support the transformation of PMADS from a reference database into a translational tool for target prioritization, drug repurposing, and personalized therapeutic design.

## Supplementary Material

gkaf1033_Supplemental_Files

## Data Availability

All data and source code supporting this study are available in Zenodo (DOI: 10.5281/zenodo.17175336), with additional resources accessible at https://pmads-db.org/download/ and https://github.com/WangHYLab/PMADS.
