# Supplementary material for: PMADS: an integrated database of curated and proteomics-inferred associations between protein post-translational modifications and drug sensitivity
Source: Nucleic Acids Res. 2025 Oct 16;54(D1):D1569–78. doi: 10.1093/nar/gkaf1033 (PMC12807662; doi:10.1093/nar/gkaf1033)

Legend of Supplementary Figures and Tables

Supplementary Figure 1. Cartoon visualization of a curated PTM–drug–disease ternary association.

Each diagram depicts a mechanistic relationship extracted from the literature, with PTM (pink), drug (grey), and disease (blue) components. Directional arrows represent regulatory flow, and labels indicate the observed modulation (e.g., PTM downregulation and disease aggravation). The visual layout reflects one of three typical patterns: (1) PTM alters drug efficacy in a disease context (e.g., acc0187), (2) the drug perturbs the PTM level, which affects disease progression (e.g., acc0177), or (3) the drug simultaneously modulates both the PTM and disease independently (e.g., acc0160).

Supplementary Table 1. Comparison of PTM-related resources.

Summary statistics and comparison of PMADS and other PTM database resources.

Supplementary Table 2. Confidence score.

Explanation of confidence scores for records in PMADS, including curated and inferred records.

Supplementary Table 3. Resource statistics.

Statistics on the quantity and categories of other data resources integrated or referenced within the PMADS database.

Supplementary Table 4. Summary statistics of the curated entries of the PMADS database.

For each major data field in the PMADS database, the table reports the relative proportions (%) of the four most frequent entries. The remaining less frequent entries are grouped and summarized as “Other”.

Supplementary Figure 1

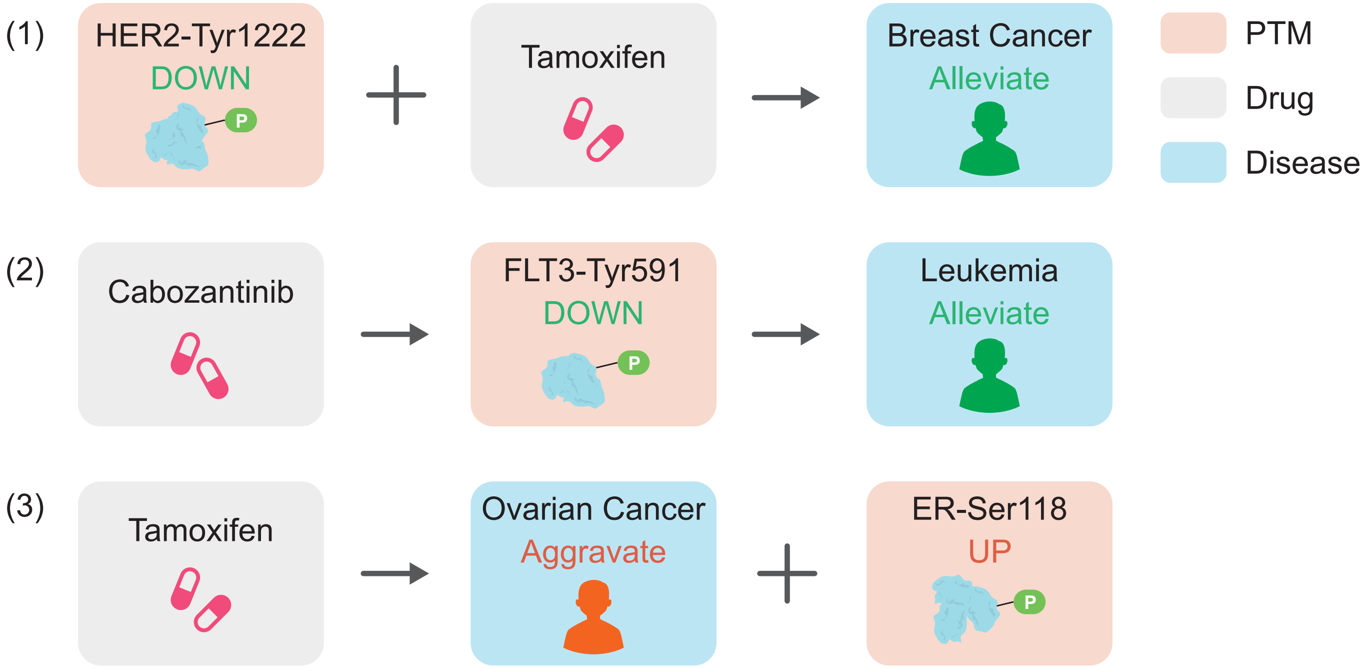

Supplement: gkaf1033_Supplemental_Files [file gkaf1033_supplemental_files.zip › Supplement Figure and Legends.pdf]
